# Tomato transcriptomic response to *Tuta absoluta* infestation

**DOI:** 10.1186/s12870-021-03129-9

**Published:** 2021-08-04

**Authors:** Daniela D’Esposito, Daniele Manzo, Alessandro Ricciardi, Antonio Pietro Garonna, Antonino De Natale, Luigi Frusciante, Francesco Pennacchio, Maria Raffaella Ercolano

**Affiliations:** 1 Department of Agricultural Sciences, University of Naples “Federico II”, Via Università 100, Portici, 80055 Naples, Italy; 2 Department of Biology, University of Naples “Federico II”, Monte Sant’ Angelo, Via Cinthia 26, 80126 Naples, Italy

**Keywords:** Comparative transcriptomics, Herbivore, Plant defense, *S. lycopersicum*, South America pinworm (*Tuta absoluta*), Trichomes, Fitness

## Abstract

**Background:**

The South America pinworm, *Tuta absoluta*, is a destructive pest of tomato that causes important losses worldwide. Breeding of resistant/tolerant tomato cultivars could be an effective strategy for *T. absoluta* management but, despite the economic importance of tomato, very limited information is available about its response to this treat. To elucidate the defense mechanisms to herbivore feeding a comparative analysis was performed between a tolerant and susceptible cultivated tomato at both morphological and transcriptome level to highlight constitutive leaf barriers, molecular and biochemical mechanisms to counter the effect of *T. absoluta* attack.

**Results:**

The tolerant genotype showed an enhanced constitutive barrier possibly as result of the higher density of trichomes and increased inducible reactions upon mild infestation thanks to the activation/repression of key transcription factors regulating genes involved in cuticle formation and cell wall strength as well as of antinutritive enzymes, and genes involved in the production of chemical toxins and bioactive secondary metabolites.

**Conclusions:**

Overall, our findings suggest that tomato resilience to the South America pinworm is achieved by a combined strategy between constitutive and induced defense system. A well-orchestrated modulation of plant transcription regulation could ensure a trade-off between defense needs and fitness costs. Our finding can be further exploited for developing *T. absoluta* tolerant cultivars, acting as important component of integrated pest management strategy for more sustainable production.

**Supplementary Information:**

The online version contains supplementary material available at 10.1186/s12870-021-03129-9.

## Background

The tomato (*Solanum lycopersicum L.*) is attacked by a wealth of insect herbivores, which cause significant crop losses and generate the need to define effective pest management programs, both under field and glasshouse conditions [1]. Among these arthropod pests, the South America pinworm, *Tuta absoluta* (Meyrick) (Lepidoptera: Gelechiidae), is one of the main threats worldwide [2], which is native from South America and, in the last decade, has invaded Europe, Africa and Asia [3].

Any aerial part of tomato plants can be attacked by *T. absoluta,* at any developmental stage. Adults usually lay eggs preferentially on the tender leaves and, to a lesser extent, on stems and fruits. Newly hatched larvae penetrate into the plant tissues, on which they feed generating mines and galleries [4]. Both yield and fruit quality can be significantly reduced by insect direct damage and by secondary pathogens, which may enter the plant tissues through the feeding wounds [5]. Since the early 1980s, chemical control was of pivotal importance in most integrated pest management (IPM) programs [6], causing several problems. Indeed, this has promoted a rapid development of insecticide resistance and number of negative effects on environment, non-target species and beneficial insects, impacting both food safety and economic sustainability of the production process [7, 8]. Biological control based on the use of predatory mirid bugs for the control of *T. absoluta* in tomatoes was successful in European horticultural pest management programs but its application in North America and some Asian countries, such as Japan, is much lower [9]. In addition, the practical use of volatiles as inducers of plant defense mechanism showed to be promising tool in pest management in tomatoes under greenhouse conditions [10]. Therefore, it would be highly desirable to integrate the use of resistant/tolerant tomato varieties in IPMs programs aimed to reduce the use of chemical and pests damage.

Tomato was extensively used as a model plant for resistance/tolerance studies and important progresses were made through both genetic and biotechnological approaches [11, 12]. It is known that tomato plants have developed numerous defense mechanisms that are effective against arthropod pests, such as physical barriers (i.e. glandular trichomes) [13], synthesis of specific metabolites [14], expression of defense proteins such as proteinase inhibitors [15] and polyphenol oxidase [16]. However, despite the economic importance of tomato, little is known about its molecular interaction with *T. absoluta*. To date, only limited information about resistance traits to South America pinworm is available for wild tomato species, such as *S. habrochaites* and *S. pennellii* [5, 17–20] Therefore, filling this research gap is crucial to obtain new tomato varieties resistant/tolerant to *T. absoluta*.

In this study, we performed a comparative analysis between a tolerant and a susceptible tomato line to unravel the plant tolerance mechanisms both at phenotypic and transcriptomic level. We examined morphological leaf traits, such as trichome types and distribution, and assessed the transcriptional reprogramming of these two tomato genotypes in response to *T. absoluta* feeding. The gathered information is a valuable basis on which to develop future breeding plans aiming to enhance the plant defense barriers against this pest.

## Methods

### Plant material and semi-field infestation trial

A cherry type tomato (*Solanum Lycopersicum*) line putatively tolerant/partially resistant to *T. absoluta*, BR221 (from now on named as ‘T’) and a susceptible one, PS650 (from now on named as ‘S’), kindly provided by FARAOO S.r.l., were used in this work. The FARAOO S.r.l., company also provided the facilities to conduct experiments according to Italian legislation and ensured the formal identification of the samples.

An infestation experiment with *T. absoluta* adults was set up in a flying nylon tunnel (90 cm × 500 cm × 90 cm) located in a glasshouse. Environmental conditions recorded in the glasshouse in which the tunnel was located were, RH = 60–70% and T° = 25 ± 2 °C. The tunnel was divided in two sections by a plastic mesh screen. For both tomato genotypes, 20 plants, 4 weeks old were transplanted in 14 cm diameter pots filled with mixed potting soil and placed in each half-tunnel, using a randomized complete block design. The plants of one half-tunnel were exposed to 100 females and 100 males of *T. absoluta,* obtained from *pupae reared on the tomato cultivar San Marzano nano,* while plants of the second half-tunnel, not exposed to *T. absoluta*, served as control. Plants were inspected for egg laying and infestation level at 10–12 day interval up to 40 days after adult release, when the overall plant damage was assessed (type of leaflet damage, the number of injuries/mines/cm^2^ of leaflets, the percentage of infested fruits), using the scoring method proposed by Resende et al*.* [17]. On that occasion, leaves with and without mines from each plant were singly collected and immediately frozen in liquid nitrogen for subsequent molecular analyses. The data obtained were processed using the software Statgraphics PlusR (Statgraphics Technologies Inc., The Plains, VA, USA). Data satisfying conditions of normality and homoscedasticity, or after appropriate transformation, if needed, were analyzed by a two-tailed Student’s test and the means were separated at the 0.001 level of significance.

### Microscopy analysis

Leaves were randomly harvested from T and S control tomato plants. Five fully expanded leaves were selected from each plant and five measurements were made on each sample. The different types of trichomes present on the two tomato genotypes, respectively belonging to classes II-VII, were identified and counted according to Channarayappa et al. [82]. Observations were made with a metallurgical microscope (Leitz Wetzlar Ortholux Microscope) equipped with an Ultropak® objective 6.5x, with a visual field area of 3.1 mm^2^. The data obtained were analyzed by the Student’s t-test (*P* < 0.001).

### Transcriptomic analysis

#### RNA extraction and sequencing

Leaves collected from single plant belonging to each treatment (Tni = Tolerant non infested; Ti = Tolerant infested; Sni = Susceptible non infested; Si = Susceptible infested), after 40 days, were pooled in independent replicates, each consisting of leaves deriving from five plants, for reducing sampling error. Three biological replicas were used for total RNA extraction, performed on frozen tomato leaves, collected from infested plants but not directly damaged by *T. absoluta*, using Spectrum™ Plant Total RNA Kit (Sigma Aldrich), according to manufacturer’s protocol. A treatment with On-Coloumn DNaseI Digestion Set (Sigma Aldrich) was carried out on total RNA to remove the contaminating genomic DNA. RNA purified samples were quantified by NanoDrop ND-1000 Spectrophotometer (Nano-Drop Technologies, Wilmington, DE, USA), RNA integrity was first checked by horizontal electrophoresis on a 1% (w/v) agarose gel with GelRed Nucleic Acid Stain 10,000X (Biothium) by UV light (UV Gel Doc BIORAD) and confirmed by Bioanalyzer (Agilent Technologies). Samples with required standards of quality were first converted to cDNA libraries and then sequenced on Illumina HiSeq1500 platform by paired-end 100 base pair (bp) sequencing using a strand-specific library. The quality of the sequences generated were evaluated by the FastQC software [21]. High quality reads, with a minimum length of 25 bp and a quality score of 35 were used for further analysis. High quality reads were aligned against the *Solanum lycopersicum* reference genome sequence (S_lycopersicum_chromosomes 2.50, [22, 23]) with TopHat (version 2.0.11). Uniquely mapping reads were used as input for Feature Counts (Subread package, version 1.4.4, [24]) to calculate gene expression values (read counts). Differentially expressed genes (DEGs) identification was performed using the edgeR package [25] considering all the genes passing the HTSFilter step.

#### Transcriptome data analysis

The annotation of biological information related to the identified differential expressed loci was performed using ITAG2.4 protein functional annotation file. The R package topGO [26] was used to carry out a Gene Ontology Enrichment Analysis with a *P*-value cut-off of 0.05. DEGs assignment to specific metabolic pathways was performed using MapMan 3.0.0 tool [27] and Plant MetGenMAP package [28]. The Sol Genomics database [23] was queried to gather additional information on annotated genes, while SolCyc database[29] was used to obtain detailed information on pathways and biochemical reactions involved in the tomato-*T. absoluta* interaction.

## Results

### Inspection of *Tuta absoluta* infestation

At all sampling times the parameters scored showed highly significant differences between the T and S lines (*P* < 0.0001, t-test, Table 1). At 10 days after adult release (10 DAAR), the difference in number of eggs/plant laid on the two tomato lines was highly significant (t = -6.16; *n* = 20; *P* < 0.001) with the average value registered on T genotype about 4 times lower than on S (Table 1). At 20 DAAR, the highly significant difference in the average number of mines/plant recorded in T and S remained in the same range (t = -5.05; *n* = 20; *P* < 0.001). The plant damage assessed 20 days later (i.e. 40 DAAR) followed the same trend of significant differences between the two genotypes, both in terms of number and type of mines. In particular, an average of 0.2 mines/cm^2^ and 0.71 mines/cm^2^ of leaflets were counted on T and S, respectively (t = -9.26; *n* = 20; *P* < 0.001). Moreover, while in the T genotype the large majority of mines were single and non-coalescent, for the S genotype this type of mine was recorded only on 52.8% of analyzed samples (t = -7.94; *n* = 20; *P* < 0.001). The difference in the level of fruit infestation per plant was even more pronounced, with a 6.5% for T genotype and 33% for S genotype (t = -7.75; *n* = 20; *P* < 0.001).

**Table 1:** Comparison of *Tuta absoluta* infestation parameters (mean ± SD) between tolerant and susceptible lines of *Solanum lycopersicum* at different days after adult release

Observed parameters and sampling time	T	S
Eggs/plant (10 DAAR)	5.7 ± 3.89	***25.3 ± 14.78
mines/plant (20 DAAR)	9.6 ± 5.95	***28.1 ± 15.25
mines/cm^2^ (40 DAAR)	0.20 ± 0.07	***0.71 ± 0.23
% single mines (40 DAAR)	52.3 ± 11.82	***88.4 ± 14.89
n. infested fruits (40 DAAR)	6.5 ± 4.28	***33 ± 14.88

*T* Tolerant, *S* Susceptible, *DAAR* days after adult release, *n* number, *SD* standard deviation, *S* mean values denoted with asterisks are significantly different from mean values recorded for T at each sampling time (*P* < 0.001)

### Leaf trichome analysis

**Fig. 1:**
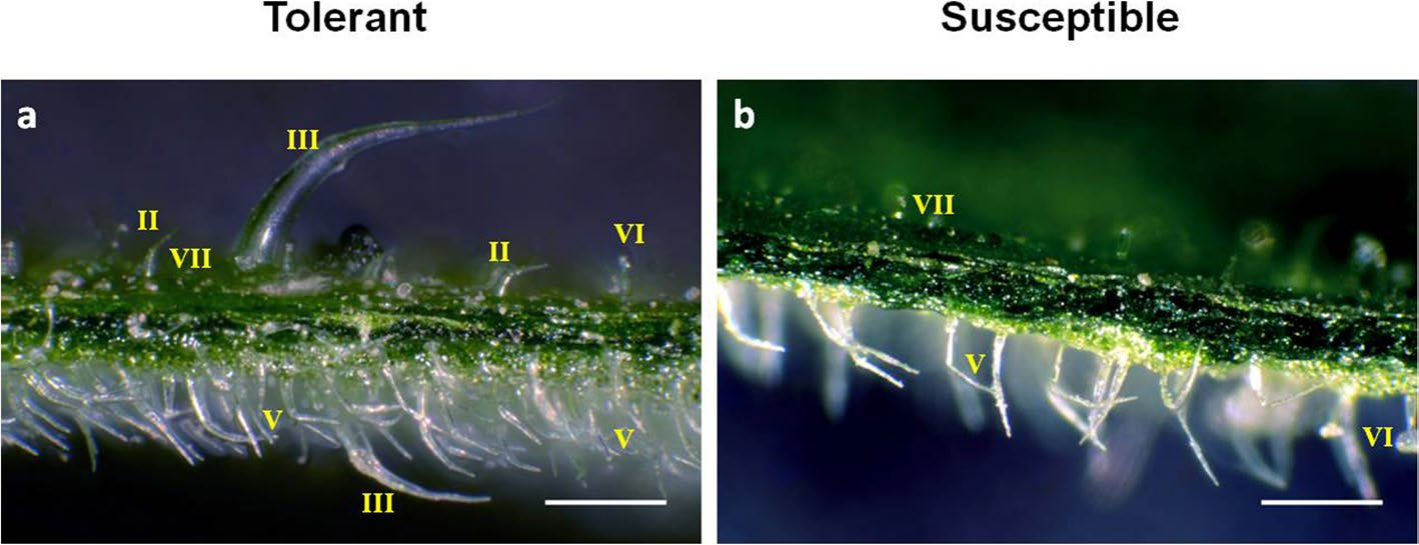
Trichomes in section of *Solanum lycopersicum*. **a** and **b** show the trichome density on leaf abaxial and adaxial sides of Tolerant and Susceptible genotypes, respectively. White bars represent 500 μm. The different types of trichomes are marked in the picture

The density of glandular and non-glandular trichomes on leaves of non-infested T and S lines showed significant differences (Fig. 1a,b; Table 2). In particular, T had a remarkable higher density of glandular trichomes types IV (adaxial) (t = -8.33; *P* < 0.001) and VI (adaxial and abaxial) (t = -6.45; *P* < 0.001 and t = -13.42; *P* < 0.001), as well as non-glandular trichomes types II (adaxial) (t = -6.96; *P* < 0.001) and V (adaxial and abaxial) (t = -6.60; *P* < 0.001 and t = -42.78; *P* < 0.001). On the contrary density of type VII glandular trichomes was significantly higher in S (t = 3.43; *P* < 0.001).

**Table 2:** Glandular and not-glandular trichomes density quantification on *Solanum lycopersicum* leaves for the adaxial and abaxial sides

	Genotype	**T**	**S**	**T**	**S**	**T**	**S**	**T**	**S**	**T**	**S**	**T**	**S**
Leaf	Trichome	**II**		**III**		**IV**		**V**		**VI**		**VII**	
Adaxial	N° (cm^2^)	15.92	0.4	11.54	7.17	108.68	20.7	142.91	43.79	82.4	20.3	33.44	86.78
	SD	19.63	3.56	17.73	23.21	89.17	31.28	126.17	46.27	76.63	39.33	36.15	48.07
	SE	2.19	0.40	1.98	2.59	9.97	3.50	14.11	5.17	8.57	4.40	4.04	5.37
	t-test	***		ns		***		***		***		***	
Abaxial	N° (cm^2^)	8.36	11.94	19.51	25.08	12.34	16.72	2367.44	587.98	238.06	74.84	9.16	78.42
	SD	20.11	22.87	26.08	28.07	21.79	30.39	276.75	248.65	85.24	67.62	35.18	176.99
	SE	2.25	2.56	2.92	3.14	2.44	3.40	30.94	27.80	9.53	7.56	3.93	19.79
	t-test	ns		ns		ns		***		***		***	

*T* Tolerant, *S* Susceptible, *SD* standard deviation, *SE* standard error, *N* number, *t-test* Student’s t-test results, *ns* not significant differences between T and S means; ***, significant differences between T and S means for *P* < 0.001

### Tomato transcriptional response

**Fig. 2:**
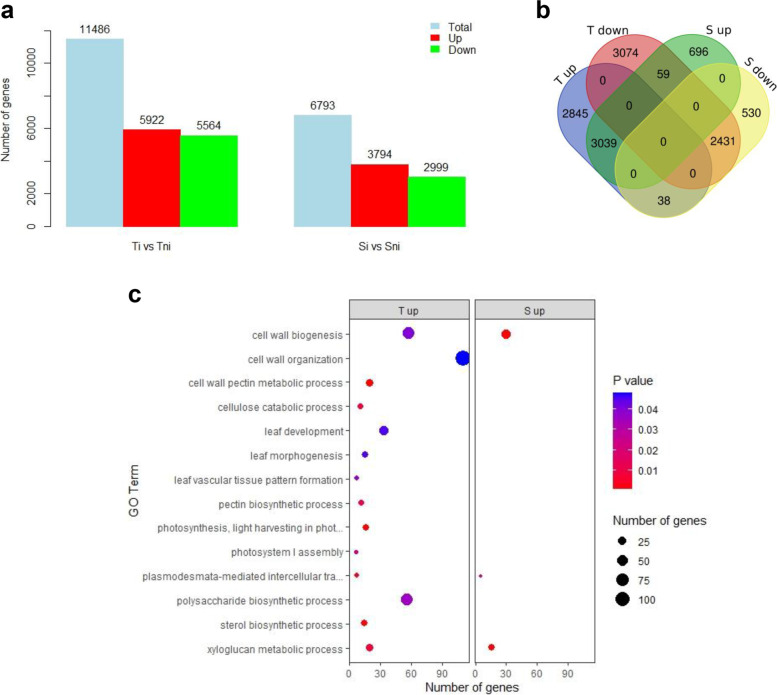
DEG analysis in tomato Tolerant and Susceptible genotypes following *T. absoluta* feeding*.***a** Number of differentially expressed genes (DEGs) detected in infested (i) vs non-infested (ni) conditions in Tolerant (T) and Susceptible (S) genotypes. **b** Venn Diagram representing the unique and common up-regulated and down-regulated DEGs among the two genotypes tested. **c** Gene ontology (GO) terms enrichment analysis at *P* < 0.05 for up-regulated genes. Circle sizes represent the number of genes included in the GO term while the color indicates the *P* value for the enrichment

The changes of gene expression in tomato leaves as affected by *T. absoluta* feeding on T and S genotypes were determined. RNA-sequencing produced an average of 24 million reads per sample (Additional File 1: Table S1). Approximatively 19,000 and 21,500 transcripts, for T and S respectively (Additional File 1: Figure S1a,b), were mapped to the tomato reference genome (SL.2.50) and used for further analysis. Differentially expressed genes (DEGs) were computed for each genotype (T and S) comparing T infested vs T non-infested (Ti vs Tni) and S infested vs S non-infested (Si vs Sni) conditions. The comparison Ti vs Tni showed a marked gene expression change in T genotype after *T. absoluta* challenge (11,486 DEGs, Fig. 2a, Additional file 2: Dataset S1-S2), while for the S genotype (Si vs Sni) a total of 6,793 DEGs were obtained (Fig. 2a, Additional file 2: Dataset S3-S4). Out of the total DEGs, 2,845, and 696 genes resulted specifically up-regulated in T and S, respectively, while 3074 in T genotype and 530 in S genotype were specifically down-regulated (Fig. 2b). More than 5,000 genes (3,039 up-regulated and 2,431 down-regulated) resulted differential expressed in both genotypes. A list of genes with the largest differences in fold changes between the two genotypes is showed in Additional File 1: Table S2.

The perturbations of tomato pathways induced by *T. absoluta* feeding were depicted by integrating Gene Ontology enrichment analysis and DEGs metabolic mapping. Key metabolic pathways, modulated during the herbivore-plant interaction, in the two tomato genotypes (T and S), in infested vs non-infested conditions, were mainly related to cell wall, sterol and steroid metabolism, leaf development and photosynthesis (Fig. 2c).

### Plant development and leaf structures morphogenesis and differentiation

**Fig. 3:**
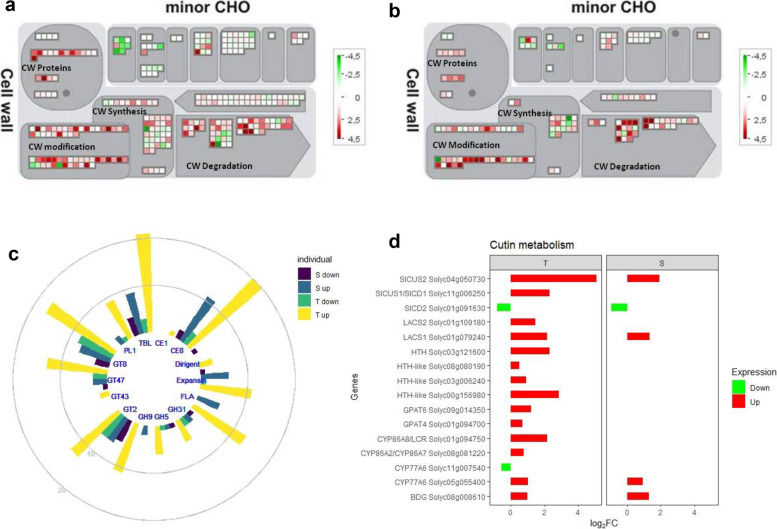
Expression data related to cell wall metabolism genes. Schematic overview of MapMan cell wall metabolism transcript abundance in infested Tolerant (**a**) and Susceptible (**b**) compared to the respective non-infested control. Differentially expressed genes (DEGs) are represented by colored squares and grouped according to MapMan functional annotation. The fold change of DEGs is indicated by the scale bar. Red indicates up-regulation, whilst green indicates down-regulation. **c** Distribution of important gene families involved in cell wall metabolism. GT, glycosyl transferases family; GH, glycoside hydrolases family; PL, polysaccharide lyases family; CE, carbohydrate esterases family; FLA, Fasciclin-like arabinogalactan. **d** Expression profiles of genes involved in cutin metabolism. X-axis indicates the log_2_ fold change (log_2_FC), Y-axis indicates selected candidate genes of DEGs. T, Tolerant, S, Susceptible

The T genotype showed an enrichment of up-regulated genes involved in leaf development, structural meristem formation and photosynthesis (Fig. 2c). The GO term “photosynthesis, light harvesting in photosystem I”, included many Chlorophyll a-b binding proteins. The GO terms “leaf development” and “leaf morphogenesis” enclosed an abundance of receptor like kinases (RLKs) such as Solyc08g061560, encoding a putative orthologue of ERECTA, Solyc08g014030, a gene closely related to the *Arabidopsis* gene encoding *SHORTROOT* (*SHR*) [30], which, together with a GRAS-domain TF, SCARECROW (SCR), regulates the duration of cell proliferation in leaves [31], the Solyc03g112750 protein tornado-TRN1 and the Solyc04g081590 CLAVATA 1, involved in meristem organization. Transcription factors (TFs) involved in meristem formation and trichome differentiation, exclusively present in T, including PHANTASTICA (Solyc09g010840) belonging to MYB family, Solyc02g092370 (SlSHRa) belonging to GRAS family, Solyc08g066500 (SlHB8) an HD-ZIP III transcription factor, and a subunit of the CAF (Solyc11g008670, FAS1) were also identified (Table 3). An enrichment of genes involved in cell wall synthesis, degradation and assembly as well in cell wall structural proteins was observed in T (Fig. 3a,b), including glycosyl transferases (GTs) families involved in cellulose (GT2), hemicelluloses (GT47), pectin (GT8) and xylan (GT43) synthesis (Fig. 3c) as well as glycoside hydrolases (GHs) belonging to GH9, GH5 and GH31 families, lyases (PL1), esterases (CE1) and expansins (Fig. 3c). Moreover, a higher number of cell wall associated genes, such as Fasciclin-like arabinogalactan (FLA) proteins (13 up-regulated in T and 5 up-regulated in S), involved in cell wall modification and assembly, and Trichome Birefringence-Like (TBL) genes (21 up-regulated in T and 9 up regulated in S) were also detected in the T genotype (Fig. 3c).

**Table 3:** List of relevant transcription factors (TFs) differentially expressed in tolerant and susceptible genotypes of *Solanum lycopersicum*

TF Family	Annotation	Gene	Ti vs Tni (log_2_FC)	Si vs Sni (log_2_FC)
**bHLH**	SlMYC1	Solyc08g005050	0.49	ns
	SlbHLH150	Solyc09g065100	1.42	-2.50
	MTB1, bHLH113	Solyc01g096050	-1.05	ns
**GRAS**	SHORTROOT (SHR)	Solyc08g014030	1.60	ns
	-	Solyc02g092370	1.20	ns
**HB-HD-ZIP**	GLABRA2 (GL2)	Solyc03g120620	0.63	-0.89
	Jasmonic acid 1	Solyc05g007180	2.32	ns
	SlHB8	Solyc08g066500	0.43	ns
**MYB/MYB-related**	MYB30	Solyc06g069850	0.78	ns
	MYB41	Solyc02g079280	-1.97	2.02
	PHANTASTICA	Solyc09g010840	1.55	ns
	TRIPTYCHON (SlTRY)	Solyc01g095640	ns	2.58
**SRS**	EXPRESSION OF TERPENOIDS 1 (EOT1)	Solyc02g062400	1.64	ns

*Ti* Tolerant infested, *Tni* Tolerant non infested, *Si* Susceptible infested, *Sni* Susceptible non infested, *ns* not significantly differentially expressed, *TF* transcription factor, *log*_*2*_*FC* log_2_-transformed fold changes

The activation of apoplast plant laccase, three dirigent-like proteins (DIR) (Solyc07G042300, Solyc10G055200, Solyc10G055230), two Pinoresinol-lariciresinol reductases, Solyc03g044720 (log_2_FC = 0.64) and Solyc06g066160 (log_2_FC = 2.17), and one cell wall peroxidase (TPX1; Solyc07g052510, log_2_FC = 3,19) suggested that the lignin/lignan synthesis was promoted in T. In addition, cutin synthesis in T genotype resulted enhanced by extensive induction of glycerol-3-phosphate acyltransferases (GPAT4, GPAT6), ω-hydroxylases, such as HOTHEAD oxidase (HTH), HTH-like, or cytochrome (Fig. 3d) and cutin monomers transport by ATP-binding cassette (ABC) transporter Solyc05g018510, orthologous to *Arabidopsis* AtABCG32.

### Perception of damage, signal transduction and defense response

**Fig. 4:**
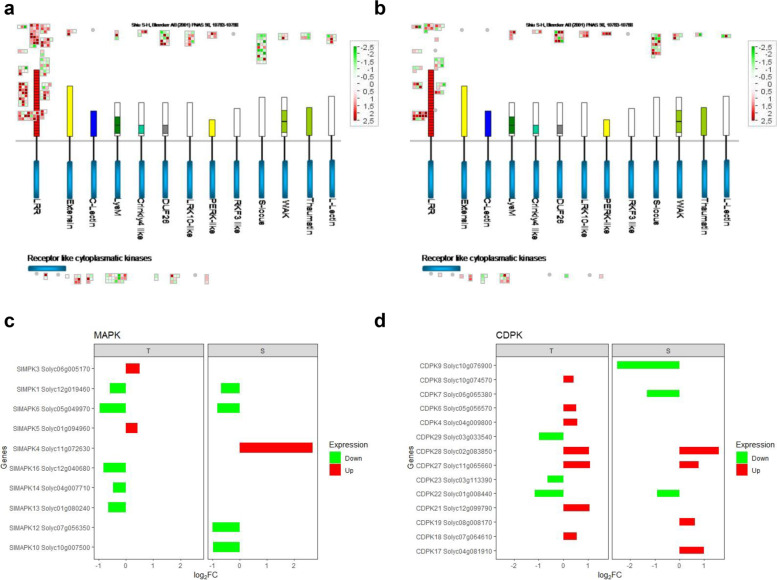
Overview of genes involved in sensing and signal transduction. **a** and **b** MapMan visualization of differentially expressed receptor-like kinase (RLK) genes in Tolerant and Susceptible genotypes, respectively. The range for the indication of up- and down-regulated genes is shown in red and green colors, respectively. The log_2_FC are shown in scale bar. **c** and **d** Expression profiles of mitogen-activated protein kinases (MAPK) and calcium-dependent protein kinases (CDPK) in T and S, respectively. X-axis indicates the log_2_ fold change (log_2_FC), Y-axis indicates selected candidate genes of DEGs. T, Tolerant; S, Susceptible

A plethora of up-regulated RLKs resulted enriched in T during *T. absoluta* challenge (Fig. 4a,b). Most of them belonged to families involved in defense response and growth. In particular, SERK1 Solyc04g072570 (LRR II), several NUCLEAR SHUTTLE PROTEIN-INTERACTING KINASE NIK members and LRR-XIII members (including Solyc03g007050-ERECTA-like 1 and Solyc08g061560-ERECTA) as well as specific extensin proteins were identified.

Divergences in the signal transduction between genotypes were also detected in the activation of mitogen-activated protein kinases (MAPKs) and calcium-dependent protein kinases (CDPKs) (Fig. 4c,d). In T was observed the up-regulation of SlMPK3, well known to be induced by wounding, and SlMAPK5, homologous to *A. thaliana* MPK4 (AT4G01370.1), a positive regulator of JA mediated signaling (Fig. 4c). Interestingly, in T also the up-regulation of SlCDPK18 (equivalent to LeCDPK1) and SlCDPK4/LeCDPK2 (Fig. 4d) was noted.

A commonly used marker for the wounding/insect response, a threonine deaminase (TD, Solyc09g008670, log_2_FC = 1,73), was exclusively produced in T, as well as the polyphenol oxidase (PPO) Solyc08g074630-PPO-F (log_2_FC = 5,04), while Solyc08g074680-PPO-B was expressed three times more in T (log_2_FC = 8,52) than in S (log_2_FC = 3,32). T genotype showed also a strong up-regulation of genes encoding defense proteins related to protease inhibitors (PIs), well known to be induced by jasmonic acid. Up to 17 PIs were activated, counting a relevant number of potato inhibitors I (PI-1) such as Solyc09g084470 (log_2_FC = 2.10), late wound-response genes induced by JA, carboxipeptidase, three metallocarboxypeptidase inhibitors (Solyc07g007260, Solyc07g007250, Solyc06g061230-putative) and two defensin proteins (Solyc07g007760, Solyc07g007750). Although Solyc07g007760 was up-regulated both in S and T, another defensin Solyc07g007710 was down-regulated (log_2_FC = -2.61) in S.

### Defensive secondary metabolites synthesis

**Fig. 5:**
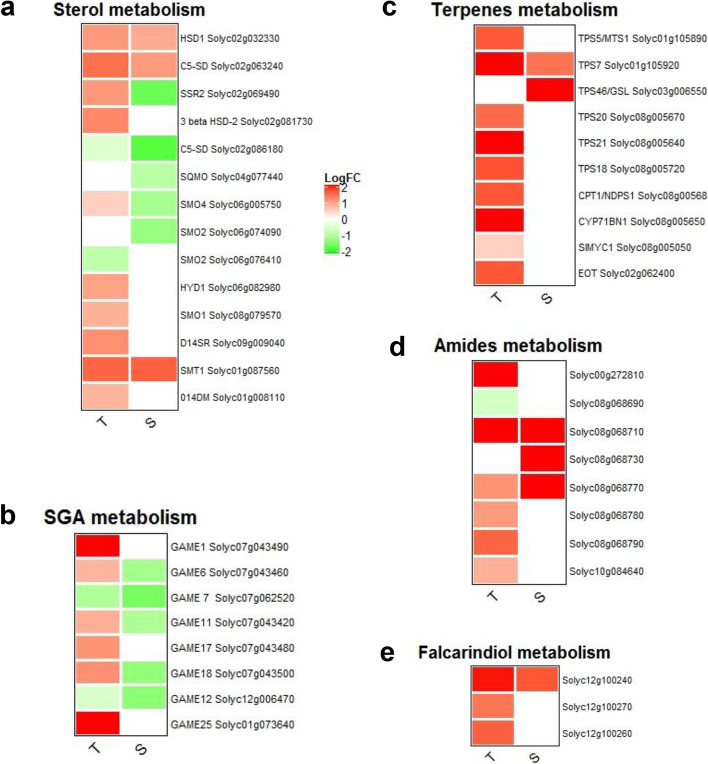
Differentially expressed genes involved in secondary metabolism of Tolerant (T) and Susceptible (S) genotypes. Up-regulated genes are shown in red, whilst down-regulated genes are in green. **a** Expression profiles of genes involved in sterol cholesterol synthesis. **b** Expression patterns of genes involved in the production of steroidal glycoalkaloids (SGA). **c** Expression analysis of genes involved in terpene synthesis. **d** Expression patterns of genes involved in amide production. **e** Expression profiles of falcarindiol biosynthesis genes

The sterol metabolism resulted strongly activated in the T genotype, as indicated by the up-regulation of a sterol side chain reductase enzyme (SSR2, Solyc02g069490), catalyzing the conversion of cycloartenol (the precursor for phytosterols) to cycloartanol, the first committed step in cholesterogenesis, that, in contrast, in S was down-regulated (Fig. 5a). In addition, the core steps of sterol biosynthesis, starting from cycloartenol, resulted clearly activated in T, as shown by the specific up-regulation of Obtusifoliol 14-alpha demethylase (O14DM), a delta14-sterol reductase (D14SR) as well as of three Sterol 4-alpha-methyl-oxidases (SMO, Solyc01g091320, Solyc06g005750 and Solyc08g079570) and an Acid phosphatase-like (Solyc04g072190). Interestingly, among the SMO, the gene Solyc06g005750 was up-regulated in T and down-regulated in S (Fig. 5a). The sterol transport seemed to be activated in the T genotype, as indicated by up-regulation of two ABC(G) transporters, Solyc12g100180 and Solyc12g100190. The production of sterol glycoalkaloids (SGA) in the T genotype was promoted by up-regulation of genes involved in glycoalkaloids biosynthesis (GAME), clustering on chromosome 7 (Fig. 5b).

DEGs were identified in the pathway of glucosinolate biosynthesis in both genotypes, while a desulfoglucosinolate sulfotransferase, Solyc03g114800, was exclusively up-regulated in T. Furthermore, two 3-isopropylmalate dehydratases (Solyc03g005730 and Solyc09g090900), involved not only in leucine synthesis but also in the production of the glucosinolate methylthioalkylmalate, resulted up-regulated only in T.

Transcriptional changes in the terpenoid biosynthetic pathway of T plants were relatively higher, both in terms of DEGs number and of their log_2_FCs. Interestingly, a cluster of genes located on chromosome 8, including the terpene synthases (TPS) TPS20, TPS21 and TPS18, a Dimethylallylcistransferase CPT1/NDPS1 and the cytochrome P450-oxidoreductase CYP71BN1, were specifically up-regulated in T. In addition, in T the up-regulation of TPS5 was observed, while in S it was observed for TPS46. Both genotypes up-regulated TPS7, but with a higher induction in T (Fig. 5c).

The biosynthesis of volatile benzenoid esters, mediated by two benzoyl transferases (SlAAT3, Solyc07g049660 and SlAAT5, Solyc05g015800) producing benzylbenzoate, resulted exclusively activated in T (log_2_FC = 2,51 and log_2_FC = 2,48). Synthesis of the toxic compound quinone 1,4-dihydroxy-2-naphthoate was promoted in T, as indicated by the up-regulation of all genes involved in the biosynthetic pathway, while only one gene was activated in S. T also showed a higher induction of genes required for the synthesis of hydroxycinnamic acid tyramine amides (Fig. 5d) and of genes involved in the production of a modified fatty acid, clustering on chromosome 12 (Fig. 5e).

### Transcription Factors

**Fig. 6:**
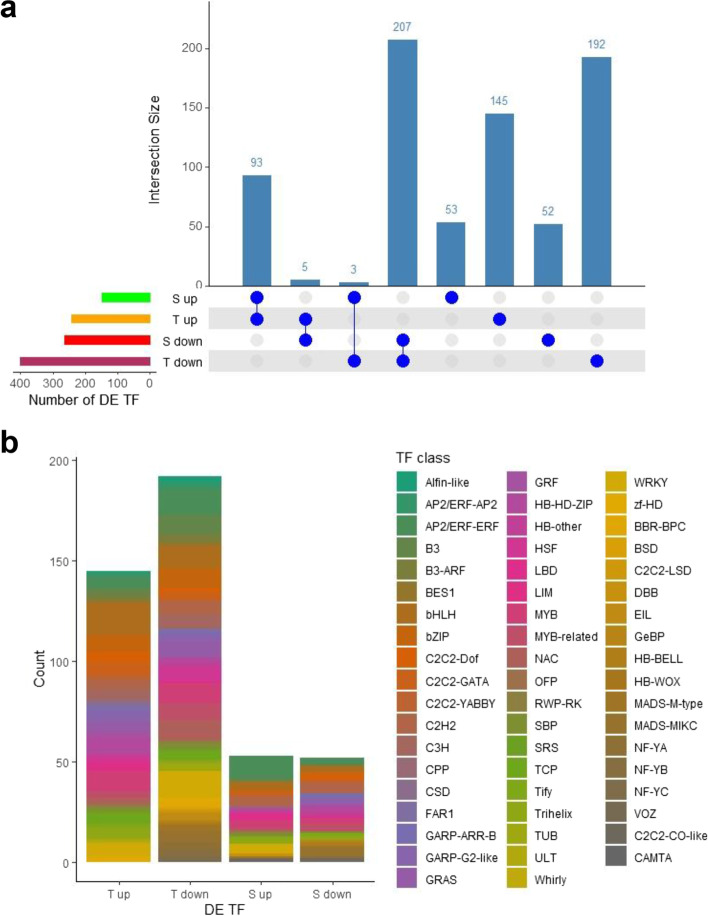
Transcription Factors analysis. **a** Visualization of intersection data regarding trascriptional factors (TFs) differentially expressed (DE). Rows indicate each dataset. S, Susceptible; T, Tolerant, up, up-regulated TFs, down, down-regulated TFs. Blue filled circle indicated set participating to the intersection. Vertical bar plots indicate the size of the intersection. **b** Classification of T and S specific TFs according to iTAK tomato database (http://itak.feilab.net/cgi-bin/itak/index.cgi)

The transcriptional regulation in response to *T. absoluta* attack mediated by TFs in T and S was examined in-depth, since T genotype showed a very high number of specifically differentially expressed TF, including 145 up-regulated and 192 down-regulated. Among the TF with a contrasting expression, five were up-regulated in T and down-regulated in S. On the contrary three were up-regulated in S and down-regulated in T (Fig. 6a). The classification of specific TF showed that they were distributed among different classes with many copies belonging to bHLH and MYB family (Fig. 6b).

Transcription factors involved in the regulation of secondary metabolites production (i.e. terpenes), trichome formation and jasmonic acid signaling, and cutin/wax metabolism were identified (Table 3). T specifically up-regulated two transcription activators, SlMYC1 and EXPRESSION OF TERPENOIDS 1 (EOT1), controlling the terpene biosynthesis in glandular trichomes and the development of type VI glandular trichome. GLABRA2 (GL2, Solyc03g120620), implicated in cell differentiation of various epidermal components, including trichomes, was also up-regulated in T and down-regulated in S. By contrast, TRIPTYCHON (SlTRY, Solyc01g095640), a negative regulator of trichome formation, was up-regulated in S while the bHLH TF (SlbHLH150, Solyc09g065100), important for marginal trichome development, was exclusively induced in T.

Jasmonic acid 1, involved in the activation of threonine deaminase (TD), was up-regulated in T (Solyc05g007180, log_2_FC = 2,32). By contrast, MTB1 (bHLH113, Solyc01g096050), that is negative regulator of JA signaling, was down-regulated in T. MYB30, a key regulator of both the protective hypersensitive response (HR) and wax biosynthesis, was up-regulated in T whilst MYB41, that mediates the negative regulation of cutin biosynthesis in response to stress, was repressed in T and induced in S. Furthermore, the negative regulator of cuticle development CFL1 (Solyc01g009770, log_2_FC = -1.01) resulted down-regulated in T.

## Discussion

### Reinforcement of external structural barriers

The first line of defense against herbivores and pathogens is often provided by physical barriers, such as trichome density and quality, cuticle tickness, and cell wall strength [32]. A higher density of glandular trichomes, (type VI and type IV) as well as non glandular trichomes (type II and V), trait of resistance to herbivory [33, 34], was observed in T. It is conceivable that difference in frequency and quality of trichomes on the surface of S compared to T could affect the *T. absoluta* feeding preference. Glandular trichomes secrete flavonoids, poisonous terpenoids and alkaloids [32, 35] that act as deterrent for oviposition and feeding of arthropod pests [36], and serve as mechanoreceptors of herbivores [37]. In particular, type VI trichomes accumulate JA, an important sensor for detecting insect movement on the leaf surface [37].

In T genotype, *T. absoluta* attack could trigger the production of trichomes on newly emerging leaves due to the up-regulation of the gene SlMYC1 involved in type VI trichome formation and in the synthesis of mono and sesquiterpenes [38]. Indeed, *SlMYC1* knocking down induced the production of smaller type VI glandular trichomes at lower densities and its knocking out led to their absence [39]. In addition, SlbHLH150 (Solyc09g065100), a transcription factor belonging to helix-loop-helix gene family [40], and GLABRA2 (GL2), encoding a homeobox leucine zipper protein, promoting leaf development and trichome formation [41–43] were also up-regulated in the T genotype. By contrast, the up-regulation in S of Solyc01g095640, a negative of regulator of trichome formation suggested that trichome morphogenesis process was reduced.

We hypothesize that the cuticle structure of T genotypes could be reinforced in response to *T. absoluta* attack thanks to the up-regulation of genes involved in cutin monomers formation and assembly, such as *HOTHEAD* (*HTH*), Glc-methanol-choline oxidoreductase family proteins [44] and *MYB30*, a crucial regulator of both wax biosynthesis and Systemic Acquired Resistance (SAR) [45]. Instead, a negative regulation of cutin biosynthesis was observed in S as indicated by the up-regulation of the genes *MYB41* and *CFL1* [46, 47]. T cell wall remodeling promoted reinforcement and the maintainance of cell function thanks to the induction of glycosyl transferases (GTs), glycoside hydrolases (GHs), polysaccharide lyases (PLs) and carbohydrate esterases (CEs). Acetylation of cell wall polymers enhanced the interaction of the hemicelluloses with other wall polymers [48], to obtain a more rigid wall conformation [49]. Moreover, the activation of a higher number of fasciclin like arabinogalactan proteins, involved in cell–cell communication during cell wall remodeling, supported the idea that changes in cell wall conformation occurred. The dirigent-like (DIR) proteins activated in T may be involved both in dynamic reorganization of the cell wall and in the production of defense compounds such as lignin and lignans [50], disruptors of insect endocrine system [51]. DIR-like protein-encoding genes during the interaction of moss *Physcomitrella patens* with *Colletotrichum gleosporioides* or *Pectobacterium carotovorum* promoted cell wall modifications, including increased incorporation of phenolic compounds [52]. In addition, the significant overexpression of the peroxidase gene *TPX1* suggested that it served as storage pool of monolignols for lignification for enhancing the rigidity and decreasing the digestibility of the cell wall [53]. The accumulation of hydroxycinnamic acid tyramine amides as well as of phenolic compounds (E-feruloyltyramine and E-p-coumaroyltyramine) in T genotype may limit the *T. absoluta* infestation as well as the digestibility of the cell wall [54, 55].

### Molecular defense response activation

The extensive reshaping of gene expression in T genotype induced by *T. absoluta* feeding activated a signaling cascade that culminated into a complex re-arrangement of primary and secondary metabolism leading to a systemic plant defense response. The cell surface perception resulted enhanced in T by the induction of cell surface receptor-kinase ERECTA (ER, Solyc03g007050), BAK1 and SERK1, involved in plant growth, developmental and immune processes regulation [56]. Members of the ER pathway interact with BAK1 in the regulation of innate immune response, plant growth and cell wall composition [57–60]. A prompter signaling in T could be mediated by the up-regulation of the calmodulin and calcium-sensing genes, involved in the activation of calcium cascade, and of MAPKs and CDPKs, such as SlMPK3 involved in systemin-mediated defense response against insect attack [61], and SlCDPK18 that modulates the wounding signaling [62]. These signal transduction pathways lead to the activation of well-characterized proteins involved in ROS detoxification such as PPO (Solyc08g074630-Solyc08g074680), proteinase and metallocarboxypeptidase inhibitors (Solyc09g084470, Solyc07g007260, Solyc07g007250, Solyc06g061230 and defensin proteins (Solyc07g007760, Solyc07g007750) that prevent pest damage [63]. Defense signaling acted synergistically to activate a finely tuned response to increase tolerance to *T. absoluta* without compromise plant fitness.

### Inducible chemical defense response amplification

Inducible defenses against herbivores include the synthesis of a wide range of species-specific toxic plant secondary metabolites (e.g., phenylpropanoids, flavonoids, anthocyanins, alkaloids, terpenoids, glucosinolates), and anti-nutritive enzymes and proteins (e.g., proteinase inhibitors (PIs), amino acid catabolizing enzymes, polyphenol oxidases, and peroxidases), mostly controlled by jasmonic acid [64–66]. T showed a much more pronounced activation of the gene arsenal involved in primary metabolism with defensive functions against insects [67], characterized by the presence of a higher number of up-regulated genes or of genes with a higher FC, such as the JA-induced defenses markers, including antinutritive and enzyme proteins TD, LAP, PPO and PI and defensins.

The divergent synthesis and composition in alkaloids, terpenes and glucosinolates between the two lines underlined the significant difference found in trichome density and type [68]. T genotype could accumulate steroidal glycoalkaloids (SGAs), as emerged from the activation of genes involved in GLYCOALKALOID METABOLISM (GAME). In particular, *GAME25* gene promoted higher levels of α-tomatine and acetoxytomatine that act synergistically against pathogens to exert cytotoxic and anti-nutritional function [69]. Moreover, genes involved in cholesterol production, that serves as the precursor for the biosynthesis of SGAs as well as of two orthologs of *Petunia hybrida PDR2*, involved in the control of steroidal compounds accumulation in leaves and trichomes [70], were highly induced in T. This is consistent with the fact that tomato resistance to phytophagus *Spodoptera litura* caterpillars was weakened by reduced SGA accumulation [71].

*T. absoluta* feeding could increase the production of some monoterpenes and other minor products in the T genotype [72] thanks to the activation of terpene synthases including the trichome-localized TPS5/MTS1, that preferentially produces linalool [73], TPS21 that, together with CYP71BN1, leads to the formation of lycosantanolol [74], and with CPT1/NDPS1 is involved in phellandrene synthesis [75]. By contrast, the up-regulation of TPS46/GLS in S lead to the production of the diterpene geranyllinalool [76]. Finally, although both genotypes up-regulated TPS7, the stronger induction in T suggests a higher production of pinene, myrcene, sabinene and linalool [77]. A different level of glucosinolate biosynthesis in T was supposed by the up-regulation of a desulfoglucosinolate sulfotransferase (Solyc03g114800) promoting the accumulation of 3-methylthiopropyl-glucosinolate at expense of other types of glucosinolates. In addition, the formation of benzylbenzoate, a major volatile compound with ovicidal properties produced after leaf disruption and wounding [78] was exclusively promoted in T by up-regulation of two benzoyl transferases (Solyc07g049660; Solyc05g015800). T genotype also showed a higher number of up-regulated genes involved in the production of 1,4-dihydroxy-2-naphthoate (DHNA), toxic precursor toward insects [79]. A major involvement of up-regulated clustered genes, required for the production of the highly functionalized oxylipin falcarindiol [80] in T leaves, was also observed. Genes involved in jasmonic acid metabolism and signaling, in the production of terpenes, glycoalkaloids and several genes in the steroid pathway as well as Sl SSR2 (Solyc02g069490), a key enzyme in the biosynthesis of toxic SGAs derived from cholesterol were up-regulated after exposure of tomato plants to volatiles resulting in enhanced resistance to *Tuta absoluta* in commercial greenhouses [10].

## Conclusions

Collectively, the gathered experimental data suggest that both constitutive barriers and inducible reactions are enhanced in T genotype in response to *T. absoluta* infestation. The South America pinworm development was impaired by multiple constraints through a consistent remodulation of the main cellular processes and secondary metabolism synthesis. Recovery from infestation occurs through photosynthesis and general cellular process promotion. A well-orchestrated modulation of transcription regulation ensured a trade-off between defense needs and fitness costs. A coordinated activation/repression of transcription factors involved in the production of antinutritive enzyme, chemical toxins and bioactive secondary metabolites drove the adjustment of plant structures and chemical compound synthesis to counter *T. absoluta* feeding.

## Supplementary Information

**Additional file 1:** **Table S1.** shows information about the raw reads quality control. **Figure S1.** shows the sequencing data filtering. **Table S2.** reports a list of genes with the largest differences in fold changes between the two genotypes.

**Additional file 2:** **Dataset S1-S2.** list the up- and down-regulated genes detected in the comparison Ti vs Tni. **Dataset S3-S4.** list the up- and down-regulated genes detected in the comparison Si vs Sni.

## Data Availability

The datasets supporting the conclusions of this article are included within the article (and its additional files). The Illumina sequence data generated during the current study are available in NCBI's Gene Expression Omnibus [81] and are accessible through GEO Series accession number GSE159085 (http://www.ncbi.nlm.nih.gov/geo).

## References

[1] Zalom FG (2003). Pests, endangered pesticides and processing tomatoes. Acta Hortic.

[2] Desneux N, Wajnberg E, Wyckhuys KAG, Burgio G, Arpaia S, Narváez-Vasquez CA (2010). Biological invasion of European tomato crops by Tuta absoluta: Ecology, geographic expansion and prospects for biological control. J Pest Sci.

[3] Campos MR, Biondi A, Adiga A, Guedes RNC, Desneux N (2017). From the Western Palaearctic region to beyond: Tuta absoluta 10 years after invading Europe. J Pest Sci.

[4] Pereyra PC, Sánchez NE (2006). Effect of two solanaceous plants on developmental and population parameters of the tomato leaf miner, Tuta absoluta (Meyrick) (Lepidoptera: Gelechiidae). Neotrop Entomol.

[5] Oliveira FA, da Silva DJH, Leite GLD, Jham GN, Picanco M (2009). Resistance of 57 greenhouse-grown accessions of Lycopersicon esculentum and three cultivars to Tuta absoluta (Meyrick)(Lepidoptera: Gelechiidae). Sci Hort.

[6] Souza JC, Reis PR (1986). Controle da traça do tomateiro em minas Gerais. Pesq agropec bras.

[7] Cuthbertson AGS, Mathers JJ, Blackburn LF, Korycinska A, Luo W, Jacobson RJ (2013). Population development of Tuta absoluta (Meyrick) (Lepidoptera: Gelechiidae) under simulated UK glasshouse conditions. Insects.

[8] Ponti L, Gilioli G, Biondi A, Desneux N, Gutierrez AP (2015). Physiologically based demographic models streamline identification and collection of data in evidence-based pest risk assessment. EPPO Bull.

[9] Pérez-Hedo M, Riahi C, Urbaneja A (2021). Use of zoophytophagous mirid bugs in horticultural crops: Current challenges and future perspectives. Pest Manag Sci.

[10] Pérez-Hedo M, Alonso-Valiente M, Vacas S (2021). Plant exposure to herbivore-induced plant volatiles: a sustainable approach through eliciting plant defenses. J Pest Sci.

[11] Ercolano MR, Sanseverino W, Carli P, Ferriello F, Frusciante L (2012). Genetic and genomic approaches for R-gene mediated disease resistance in tomato: Retrospects and prospects. Plant Cell Rep.

[12] Cappetta E, Andolfo G, Di Matteo A, Barone A, Frusciante L, Ercolano MR (2020). Accelerating tomato breeding by exploiting genomic selection approaches. Plants.

[13] Simmons AT, Gurr GM (2005). Trichomes of Lycopersicon species and their hybrids: Effects on pests and natural enemies. Agric For Entomol.

[14] Carter CD, Sacalis JN, Gianfagna TJ. Zingiberene and resistance to Colorado potato beetle in Lycopersicon hirsutum f. hirsutum. J. Agric. Food Chem. 1989;37(1): 206–210.

[15] Howe GA, Lightner J, Browse J, Ryan CA (1996). An octadecanoid pathway mutant (JL5) of tomato is compromised in signaling for defense against insect attack. Plant Cell.

[16] Thaler JS (1999). Induced resistance in agricultural crops: Effects of jasmonic acid on herbivory and yield in tomato plants. Environ Entomol.

[17] Vilela De Resende JT, Maluf WR, Faria MV, Pfann AZ, Rodrigues Do Nascimento I. Acylsugars in tomato leaflets confer resistance to the South American tomato pinworm, Tuta absoluta Meyr. Sci Agric. 2006;63(1):20–5.

[18] Pereira GVN, Maluf WR, Gonçalves LD, do Nascimento IR, Gomes LAA, Licursi V. Selection towards high acylsugar levels in tomato genotypes and its relationship with resistance to spider mite (Tetranychus evansi) and to the South American pinworm (Tuta absoluta). Cienc e Agrotecnologia. 2008;32(3):996–1004.

[19] Campos ML, De Almeida M, Rossi ML, Martinelli AP, Litholdo Junior CG, Figueira A (2009). Brassinosteroids interact negatively with jasmonates in the formation of anti-herbivory traits in tomato. J Exp Bot.

[20] Maluf WR, Maciel GM, Gomes LAA, Cardoso M das G, Gonçalves LD, da Silva EC, et al. Broad-spectrum arthropod resistance in hybrids between high-and low-acylsugar tomato lines. Crop Sci. 2010;50(2):439–50.

[21] Babraham Bioinformatics.http://www.bioinformatics.babraham.ac.uk/projects/fastqc/.

[22] The Tomato Genome Consortium., Kazusa DNA Research Institute., Sato, S. et al. The tomato genome sequence provides insights into fleshy fruit evolution. Nature 2012;485, 635–641. 10.1038/nature11119 PMC3378239 22660326

[23] Sol Genomics Network. https://solgenomics.net.

[24] Liao Y, Smyth GK, Shi W (2014). FeatureCounts: An efficient general purpose program for assigning sequence reads to genomic features. Bioinformatics.

[25] Robinson MD, McCarthy DJ, Smyth GK (2009). edgeR: A Bioconductor package for differential expression analysis of digital gene expression data. Bioinformatics.

[26] Alexa A, Rahnenfuhrer J. Gene set enrichment analysis with topGO. 2018.

[27] Thimm O, Bläsing O, Gibon Y, Nagel A, Meyer S, Krüger P (2004). MAPMAN: A user-driven tool to display genomics data sets onto diagrams of metabolic pathways and other biological processes. Plant J.

[28] Joung J-G, Corbett AM, Fellman SM, Tieman DM, Klee HJ, Giovannoni JJ, et al. Plant MetGenMAP: An Integrative Analysis System for Plant Systems Biology. Plant Physiol. 2009;151(4):1758–68. Available from: http://www.plantphysiol.org/cgi/doi/10.1104/pp.109.145169 10.1104/pp.109.145169 PMC2786002 19819981

[29] SolCyc Biochemical Pathways. https://solgenomics.net/pages/solcyc/.

[30] Huang W, Xian Z, Kang X, Tang N, Li Z (2015). Genome-wide identification, phylogeny and expression analysis of GRAS gene family in tomato. BMC Plant Biol.

[31] Dhondt S, Coppens F, De Winter F, Swarup K, Merks RM, Inzé D, Bennett MJ, Beemster GT (2010). SHORT-ROOT and SCARECROW regulate leaf growth in Arabidopsis by stimulating S-phase progression of the cell cycle. Plant Physiol.

[32] War AR, Paulraj MG, Ahmad T, Buhroo AA, Hussain B, Ignacimuthu S (2012). Mechanisms of Plant Defense against Insect Herbivores. Plant Signal Behav.

[33] Kennedy GG. Tomato, Pests, Parasitoids, and Predators: Tritrophic Interactions Involving the Genus Lycopersicon. Annu Rev Entomol. February 2003;2003(48):51–72. 10.1146/annurev.ento.48.091801.112733 12194909

[34] Kivimäki M, Kärkkäinen K, Gaudeul M, Løe G, Ågren J (2007). Gene, phenotype and function: GLABROUS1 and resistance to herbivory in natural populations of Arabidopsis lyrata. Mol Ecol.

[35] Glas JJ, Schimmel BCJ, Alba JM, Escobar-Bravo R, Schuurink RC, Kant MR (2012). Plant glandular trichomes as targets for breeding or engineering of resistance to herbivores. Int J Mol Sci.

[36] Handley R, Ekbom B, Ågren J (2005). Variation in trichome density and resistance against a specialist insect herbivore in natural populations of Arabidopsis thaliana. Ecol Entomol.

[37] Peiffer M, Tooker JF, Luthe DS, Felton GW (2009). Plants on early alert: Glandular trichomes as sensors for insect herbivores. New Phytol.

[38] Xu J, Van Herwijnen ZO, Dräger DB, Sui C, Haring MA, Schuurink RC (2018). SlMYC1 regulates type VI glandular trichome formation and terpene biosynthesis in tomato glandular cells. Plant Cell.

[39] Flood PJ (2019). The smell of transcription: The slmyc1 transcription factor makes tomato plants smelly. Plant Cell.

[40] Sun H, Fan HJ, Ling HQ. Genome-wide identification and characterization of the bHLH gene family in tomato. BMC Genomics. 2015;16(1). 10.1186/s12864-014-1209-2 PMC4312455 25612924

[41] Ohashi Y, Oka A, Ruberti I, Morelli G, Aoyama T (2002). Entopically additive expression of GLABRA2 alters the frequency and spacing of trichome initiation. Plant J.

[42] Gao Y, Gong X, Cao W, Zhao J, Fu L, Wang X (2008). SAD2 in arabidopsis functions in trichome initiation through mediating GL3 function and regulating GL1, TTG1 and GL2 expression. J Integr Plant Biol.

[43] D’Esposito D, Cappetta E, Andolfo G, Ferriello F, Borgonuovo C, Caruso G, et al. Deciphering the biological processes underlying tomato biomass production and composition. Plant Physiol Biochem. 2019;143(August):50–60. Available from: 10.1016/j.plaphy.2019.08.010 10.1016/j.plaphy.2019.08.010 31479882

[44] Krolikowski KA, Victor JL, Wagler TN, Lolle SJ, Pruitt RE (2003). Isolation and characterization of the Arabidopsis organ fusion gene HOTHEAD. Plant J.

[45] Raffaele S, Vailleau F, Léger A, Joubès J, Miersch O, Huard C (2008). A MYB transcription factor regulates very-long-chain fatty acid biosynthesis for activation of the hypersensitive cell death response in Arabidopsis. Plant Cell.

[46] Cominelli E, Sala T, Calvi D, Gusmaroli G, Tonelli C (2008). Over-expression of the Arabidopsis AtMYB41 gene alters cell expansion and leaf surface permeability. Plant J.

[47] Wu R, Li S, He S, Waßmann F, Yu C, Qin G (2011). CFL1, a WW domain protein, regulates cuticle development by modulating the function of HDG1, a class IV homeodomain transcription factor, in rice and arabidopsis. Plant Cell.

[48] Busse-Wicher M, Gomes TCF, Tryfona T, Nikolovski N, Stott K, Grantham NJ, Dupree P (2014). The pattern of xylan acetylation suggests xylan may interact with cellulose microfibrils as a twofold helical screw in the secondary plant cell wall of Arabidopsis thaliana. Plant J.

[49] Gao Y, He C, Zhang D, Liu X, Xu Z, Tian Y (2017). Two trichome birefringence-like proteins mediate xylan acetylation, which is essential for leaf blight resistance in rice. Plant Physiol.

[50] Paniagua C, Bilkova A, Jackson P, Dabravolski S, Riber W, Didi V (2017). Dirigent proteins in plants: modulating cell wall metabolism during abiotic and biotic stress exposure. J Exp Bot.

[51] Harmatha J, Dinan L (2003). Biological activities of lignans and stilbenoids associated with plant-insect chemical interactions. Phytochem Rev.

[52] Reboledo G, del Campo R, Alvarez A, Montesano M, Mara H, de León IP (2015). Physcomitrella patens activates defense responses against the pathogen Colletotrichum gloeosporioides. Int J Mol Sci.

[53] Quiroga M, Guerrero C, Botella MA, Barceló A, Amaya I, Medina MI (2000). A tomato peroxidase involved in the synthesis of lignin and suberin. Plant Physiol.

[54] Grandmaison J, Olah GM, Van Calsteren M, Furlan V (1993). Characterization and locali-zation of plant phenolics likely involved in the pathogen resistance expressed by endomycorrhizal roots. Mycorrhiza.

[55] Hagel JM, Facchini PJ (2005). Elevated tyrosine decarboxylase and tyramine hydroxycinnamoyltransferase levels increase wound-induced tyramine-derived hydroxycinnamic acid amide accumulation in transgenic tobacco leaves. Planta.

[56] He Y, Zhou J, Shan L, Meng X. Plant cell surface receptor-mediated signaling - A common theme amid diversity. J Cell Sci. 2018;131(2). 10.1242/jcs.209353 PMC6518216 29378836

[57] Llorente F, Alonso-Blanco C, Sánchez-Rodriguez C, Jorda L, Molina A (2005). ERECTA receptor-like kinase and heterotrimeric G protein from Arabidopsis are required for resistance to the necrotrophic fungus Plectosphaerella cucumerina. Plant J.

[58] Sánchez-Rodríguez C, Estévez JM, Llorente F, Hernández-Blanco C, Jordá L, Pagán I, Berrocal M, Marco Y, Somerville S, Molina A (2009). The ERECTA Receptor-Like Kinase Regulates Cell Wall-Mediated Resistance to Pathogens in Arabidopsis thaliana. Mol Plant Microbe Interact.

[59] Jordá L, Sopeña-Torres S, Escudero V, Nuñez-Corcuera B, Delgado-Cerezo M, Torii KU (2016). ERECTA and BAK1 receptor like kinases interact to regulate immune responses in Arabidopsis. Front Plant Sci.

[60] Zhou J, Wang P, Claus LAN, Savatin DV, Xu G, Wu S, Meng X, Russinova E, He P, Shan L (2019). Proteolytic processing of SERK3/BAK1 regulates plant immunity, devel-opment, and cell death. Plant Physiol.

[61] Kandoth PK, Ranf S, Pancholi SS, Jayanty S, Walla MD, Miller W (2007). Tomato MAPKs LeMPK1, LeMPK2, and LeMPK3 function in the systemin-mediated defense response against herbivorous insects. Proc Natl Acad Sci U S A.

[62] Chico JM, Raíces M, Téllez-Iñón MT, Ulloa RM (2002). A calcium-dependent protein kinase is systemically induced upon wounding in tomato plants. Plant Physiol.

[63] Díez-Díaz M, Conejero V, Rodrigo I, Pearce G, Ryan CA (2004). Isolation and characteri-zation of wound-inducible carboxypeptidase inhibitor from tomato leaves. Phytochemistry.

[64] Erb M, Meldau S, Howe GA (2012). Role of phytohormones in insect-specific plant reac-tions. Trends Plant Sci.

[65] Grinberg-Yaari M, Alagarmalai J, Lewinsohn E, Perl-Treves R, Soroker V (2015). Role of jasmonic acid signaling in tomato defense against broad mite, Polyphagotarsonemus latus (Acari: Tarsonemidae). Arthropod Plant Interact.

[66] Erb M, Reymond P (2019). Molecular Interactions between Plants and Insect Herbivores. Annu Rev Plant Biol.

[67] Schwachtje J, Baldwin IT (2008). Why does herbivore attack reconfigure primary metabolism?. Plant Physiol.

[68] Tian D, Tooker J, Peiffer M, Chung SH, Felton GW (2012). Role of trichomes in defense against herbivores: Comparison of herbivore response to woolly and hairless trichome mutants in tomato (Solanum lycopersicum). Planta.

[69] Sonawane PD, Heinig U, Panda S, Gilboa NS, Yona M, Pradeep Kumar S (2018). Short-chain dehydrogenase/reductase governs steroidal specialized metabolites structural diversity and toxicity in the genus Solanum. Proc Natl Acad Sci U S A.

[70] Sasse J, Schlegel M, Borghi L, Ullrich F, Lee M, Liu GW (2016). Petunia hybrida PDR2 is involved in herbivore defense by controlling steroidal contents in trichomes. Plant Cell Environ.

[71] Nakayasu M, Shioya N, Shikata M, Thagun C, Abdelkareem A, Okabe Y (2018). JRE4 is a master transcriptional regulator of defense-related steroidal glycoalkaloids in tomato. Plant J.

[72] de Falco B, Manzo D, Incerti G, Garonna AP, Ercolano M, Lanzotti V (2019). Metabo-lomics approach based on NMR spectroscopy and multivariate data analysis to explore the interaction between the leafminer Tuta absoluta and tomato (Solanum lycopersicum). Phytochem Anal.

[73] Van Schie CCN, Haring MA, Schuurink RC (2007). Tomato linalool synthase is induced in trichomes by jasmonic acid. Plant Mol Biol.

[74] Matsuba Y, Zi J, Jones AD, Peters RJ, Pichersky E (2015). Biosynthesis of the diterpenoid lycosantalonol via nerylneryl diphosphate in solanum lycopersicum. PLoS ONE.

[75] Schilmiller AL, Schauvinhold I, Larson M, Xu R, Charbonneau AL, Schmidt A (2009). Monoterpenes in the glandular trichomes of tomato are synthesized from a neryl diphosphate precursor rather than geranyl diphosphate. Proc Natl Acad Sci U S A.

[76] Falara V, Alba JM, Kant MR, Schuurink RC, Pichersky E (2014). Geranyllinalool synthases in solanaceae and other angiosperms constitute an ancient branch of diterpene synthases involved in the synthesis of defensive compounds. Plant Physiol.

[77] Falara V, Akhtar TA, Nguyen TTH, Spyropoulou EA, Bleeker PM, Schauvinhold I (2011). The tomato terpene synthase gene family. Plant Physiol.

[78] Seino Y, Suzuki Y, Sogawa K (1996). An Ovicidal Substance Produced by Rice Plants in Response to Oviposition by the Whitebacked Planthopper, Sogatella furcifera (HORVATH) (Homoptera: Delphacidae). Appl Entomol Zool.

[79] Widhalm JR, Rhodes D. Biosynthesis and molecular actions of specialized 1,4-naphthoquinone natural products produced by horticultural plants. Hortic Res.. 2016;3(July). Available from: http://dx.doi.org/10.1038/hortres.2016.46 10.1038/hortres.2016.46 PMC5030760 27688890

[80] Jeon JE, Kim JG, Fischer CR, Mehta N, Dufour-Schroif C, Wemmer K, Mudgett MB, Sattely E (2020). A Pathogen- Responsive gene cluster for highly modified fatty acids in tomato. Cell.

[81] Edgar R, Domrachev M, Lash AE (2002). Gene Expression Omnibus: NCBI gene expression and hybridization array data repository. Nucleic Acids Res.

[82] Channarayappa C, Shivashankar G, Muniyappa V, Frist RH (1992). Resistance of Lycopersicon species to Bemisia tabaci, a tomato leaf curl virus vector. Can J Bot.

