# Supplementary material for: Tomato transcriptomic response to Tuta absoluta infestation
Source: BMC Plant Biol. 2021 Aug 4;21:358. doi: 10.1186/s12870-021-03129-9 (PMC8336066; doi:10.1186/s12870-021-03129-9)

**Additional file 1**

**Tomato transcriptomic response to *Tuta absoluta* infestation**

**Daniela D’Esposito*, Daniele Manzo*, Alessandro Ricciardi, Antonio Pietro Garonna, Antonino De Natale, Luigi Frusciante, Francesco Pennacchio, Maria Raffaella Ercolano**

**Supplemental Table and Supplemental Figure**

**Table S1.** Number of sequenced reads before and after quality control and mapping statistics.

| **Sample Name** | **Number of reads before data quality control** | **Number of reads after data quality control** | **Mapped reads (percentage)** | **Unmapped reads (percentage)** |
| --- | --- | --- | --- | --- |
| Tni Rep1 | 25713821 | 24390437 | 21840466 (89.54%) | 2549971 (10.46%) |
| Tni Rep2 | 19706221 | 19069911 | 16943045 (88.85%) | 2126866 (11.15%) |
| Sni Rep1 | 22224662 | 21391274 | 19194512 (89.73%) | 2196762 (10.27%) |
| Sni Rep2 | 23663488 | 22993345 | 19679194 (85.59%) | 3314151 (14.41%) |
| Ti Rep1 | 28968237 | 27930286 | 25358935 (90.79%) | 2571351 (9.21%) |
| Ti Rep2 | 30377806 | 28976601 | 26557923 (91.65%) | 2418678 (8.35%) |
| Si Rep1 | 22486053 | 21458005 | 19027372 (88.67%) | 2430633 (11.33%) |
| Si Rep2 | 27851400 | 26308810 | 23836646 (90.60%) | 2472164 (9.40%) |

**Table S2.** Table of the largest differences in Fold Change (FC) between the two genotypes.

| **Gene** | **Ti vs Tni (logFC)** | **Si vs Sni (LogFC)** | **Annotation** |
| --- | --- | --- | --- |
| Solyc04g081790 | 7,54 | 3,87 | GDSL esterase/lipase At5g42170 (GDL90_ARATH) |
| Solyc04g078140 | 2,15 | -1,56 | Cytochrome B5 (Q9ZSP7_PETHY) |
| Solyc09g065100 | 1,42 | -2,50 | Transcription factor (Q9M4A8_MAIZE) |
| Solyc01g073860 | 9,00 | 4,97 | CHP-rich zinc finger protein (C8CBB1_WHEAT) |
| Solyc12g005430 | 7,04 | 2,99 | Acyltransferase-like protein (Q589X8_TOBAC) |
| Solyc06g083900 | 6,20 | 2,01 | R2R3MYB transcription factor 13 (manually curated) |
| Solyc12g088170 | 1,54 | -2,80 | Hydroxycinnamoyl CoA quinate transferase (D6BK25_CYNSC) |
| Solyc11g071470 | 9,27 | 4,86 | Hydroxycinnamoyl CoA shikimate/quinate hydroxycinnamoyltransferase-like protein (Fragment) (B9GF60_POPTR) |
| Solyc08g080040 | 1,90 | -2,53 | Anthocyanidin synthase (D3XFG1_THECC) |
| Solyc02g085020 | 2,61 | -1,85 | dihydroflavonol 4-reductase (manually curated) |
| Solyc12g098590 | 2,64 | -1,83 | UDP-glucosyltransferase family 1 protein (C6KI42_CITSI) |
| Solyc02g081340 | 1,43 | -3,11 | Glutathione S-transferase (O24261_PETHY) |
| Solyc11g066580 | 2,48 | -2,22 | Cytochrome P450 |
| Solyc03g025190 | 2,83 | -2,00 | anthocyanin permease (manually curated) |
| Solyc07g008240 | 8,95 | 3,87 | Non-symbiotic hemoglobin protein (Q4VIX3_GOSHI) |
| Solyc02g089350 | 7,65 | 2,54 | Gibberellin regulated protein (Q2HRH3_MEDTR) |
| Solyc08g074680 | 8,52 | 3,32 | partialpolyphenol oxidase A (manually curated) |
| Solyc09g009980 | 7,74 | 2,53 | Small auxin up-regulated RNA70 (manually curated) |
| Solyc01g057080 | 8,76 | 3,11 | Ethylene-responsive transcription factor 13 (ERF99_ARATH) |
| Solyc12g009290 | 7,98 | 1,91 | SAUR33-auxin-responsive SAUR family member (B6T035_MAIZE) |
| Solyc03g113930 | -3,78 | 7,18 | class IV heat shock protein (B6T3F5_MAIZE) |
| Solyc12g013750 | -7,62 | 0,85 | Mannan endo-1,4-beta-mannosidase 3 (MAN3_SOLLC) |
| Solyc03g098100 | -4,59 | 3,61 | Reductase 2 (Q6TY50_HYDMC) |
| Solyc12g038430 | -8,64 | -1,01 | Kinesin-like calmodulin binding protein (Q9FQL7_MAIZE) |
| Solyc08g062450 | -4,63 | 2,91 | class II heat shock protein (B6T339_MAIZE) |
| Solyc03g007790 | -4,10 | 2,93 | Receptor-like protein kinase (Q9FLV4_ARATH) |
| Solyc11g071760 | -4,95 | 1,72 | regulator of gene silencing AY642285 (manually curated) |
| Solyc07g008110 | -1,56 | 4,73 | Blue copper protein (Fragment) (O82576_MAIZE) |
| Solyc01g102960 | -3,31 | 2,97 | class IV heat shock protein (B6T3F5_MAIZE) |
| Solyc01g104730 | -2,19 | 3,94 | MAPK activating protein (B6TKI4_MAIZE) |
| Solyc06g036290 | -3,64 | 2,16 | heat shock protein 90 (manually curated) |
| Solyc08g078700 | -2,17 | 3,41 | mitochondrial small heat shock protein (manually curated) |
| Solyc01g107830 | -1,54 | 3,18 | UDP-glucosyltransferase family 1 protein (C6KI43_CITSI) |
| Solyc03g095650 | -5,32 | -0,77 | MLO-like protein 17 (C6EWF0_VITVI) |
| Solyc02g094000 | -2,04 | 2,43 | Calmodulin-like protein (Q0VJ70_DATME) |
| Solyc01g096720 | 2,49 | 6,83 | Major facilitator superfamily transporter (A8NUY3_COPC7) |
| Solyc10g050970 | -2,18 | 2,06 | Ethylene responsive transcription factor 2b (C0J9I6_9ROSA) |
| Solyc10g008040 | -5,91 | -1,74 | Seed biotin-containing protein SBP65 (SBP65_PEA) |
| Solyc04g079660 | -1,62 | 2,49 | Cytochrome P450 |
| Solyc08g067630 | -5,64 | -1,53 | Unknown Protein |

**Fig. S1.** Graph showing the number of loci in the raw data, the removed loci according to HTSFilter analysis and those retained for further analysis for the Tolerant (**a**) and Susceptible (**b**) genotypes.


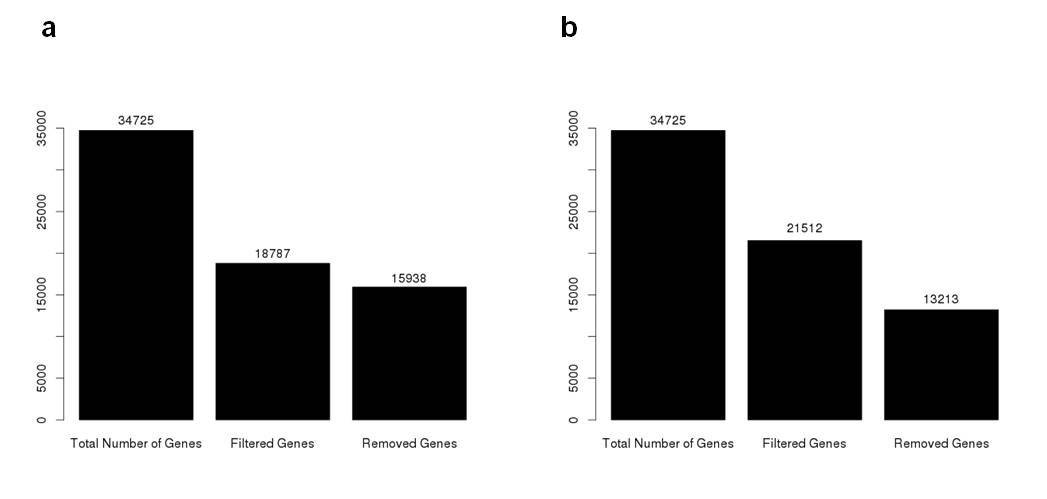

Supplement: Supplementary file 1 — Additional file 1: Table S1. shows information about the raw reads quality control. Figure S1. shows the sequencing data filtering. Table S2. reports a list of genes with the largest differences in fold changes between the two genotypes. [file 12870_2021_3129_MOESM1_ESM.docx]
